# The profile and clinical picture of children with undernutrition admitted to National District Hospital

**DOI:** 10.11604/pamj.2020.37.237.25261

**Published:** 2020-11-13

**Authors:** Hanneke Brits, Lezanne Botha, Wiseman Maakomane, Thabiso Malefane, Tayob Luthfiya, Tshepo Tsoeueamakwa, Gina Joubert

**Affiliations:** 1 Department of Family Medicine, Faculty of Health Sciences, University of the Free State, Bloemfontein, South Africa,; 2 University of the Free State, Bloemfontein, South Africa,; 3 Department of Biostatistics, School of Biomedical Sciences, Faculty of Health Sciences, University of the Free State, Bloemfontein, South Africa

**Keywords:** Undernutrition, South Africa, childhood development, intervention, low birth weight

## Abstract

**Introduction:**

undernutrition has a profound effect on growth, development and susceptibility to infectious disease. In Africa, it was found that undernutrition is an underlying factor in around 35% of the preventable deaths of children under the age of 5 years. The first 1000 days of life is most crucial for childhood development. Undernourished children in their first 1000 days of development experience a significant reduction in brain development which cannot be regained later in life. The aim was to describe the profile and clinical picture of admitted children with undernutrition, in order to identify areas for intervention.

**Methods:**

a descriptive study design with an analytical component was used. Data for undernourished admitted children, ages 2-71 months, for the study period 2016-2017 at the study site were included in the study. Data were collected from patient files and summarised by frequencies and percentages.

**Results:**

data were collected from 172 patient files, which is > 80% of all children eligible for inclusion. Most (88.0%) of the children had a weight for age < -2 SD and 18.6% had bilateral pitting oedema. More than 80% of the children were still in their first 1000 days of life, 42.8% were born with low birth weight and 24.2% were not breastfed. Head circumference was only recorded for 16.3%. Presenting symptoms were cough, fever and diarrhea - in line with those covered in the Integrated Management of Childhood Illness (IMCI).

**Conclusion:**

most children presented within the first 1000 days of life, making focussed interventions possible. Areas identified for intervention were babies with low birth weight and babies not breastfed. As most children presented with IMCI symptoms, nurses should also assess the nutritional status of these children in accordance with the guidelines. Lack of anthropometric measurements and poorly recorded feeding histories should be addressed.

## Introduction

Undernutrition is defined as “the lack of proper nutrition caused by not having enough food or not eating enough food containing substances necessary for growth and health” [1]. Undernutrition has been identified as a major contributor to child mortality [2]. According to the Child Problem Identification Programme, 42% of children aged 1-5 years and 29.8% of infants who died in a South African hospital between 2012 and 2013 were severely undernourished [3]. Evidence suggests that undernutrition in infants and children has a profound effect on growth and development as well as susceptibility to infectious disease [4].

In 2000, the United Nations developed the Millennium Development Goals (MDGs) to address poverty and mortality worldwide by 2015. Some of the goals included: eradicate extreme poverty and hunger, and reduce child mortality rates [5]. Goal 2 of the Sustainable Development goals for 2030 addresses nutrition under the heading “Zero hunger” [6]. Although huge efforts were made to reach the MDG´s, undernutrition actually worsened in parts of South Africa like the Free State province between 2010 and 2013 [7] despite nutrition intervention programmes [8].

The main causes of undernutrition are inadequate food intake, unsafe water, factors related to society, inadequate sanitation or insufficient hygiene, diseases, maternal factors, gender issues, and overall poverty [3,9]. As underweight children are more susceptible to almost all infectious diseases, the indirect disease burden of undernutrition is estimated to be an order of magnitude higher than the disease burden of the direct effects of undernutrition [10]. In the South African Bill of Rights Section 28(1) (c), it is stated that every child has the right to basic nutrition. In South Africa, 11.8% of children grow up in households with reported hunger. In the Free State, 14.8% of children are in this category [11].

A close relationship exists between HIV and undernutrition. HIV may contribute to undernutrition due poor appetite and food intake during illness, mouth ulcers (caused by opportunistic infections), the higher metabolic rate (caused by low grade fever), chronic diarrhoea and the socio-economic impact of an ill or deceased parent or carer [12]. The nutritional status of HIV infected people is compromised by the disease, which increases susceptibility to other diseases [13]. HIV can also be transmitted via breast milk. In South Africa, it was found that HIV transmission rates were similar at six months in infants who were formula fed and those exclusively breastfed for three months or more. The rate of transmission of mother-to-child was higher in the group that received mixed feeding [4].

Previous studies confirm that undernutrition is more common in children with HIV [14,15]. The Burkina Faso study used anthropometric indicators to study the relationship between nutritional status and HIV and found that HIV infected children have more nutritional requirements than uninfected children. Infection with HIV suppressed the immunity, which also contributed to undernutrition. This study also reported on poor nutrition in HIV-positive households, more nutritional requirements and co-infection that predisposed to undernutrition [14].

In a Tanzanian study, it was found that undernutrition is an underlying factor in around 35% of the preventable deaths of children under the age of five years. It was also reported that over half of children with HIV are suffering from severe undernutrition. Initiation of antiretroviral therapy in these children revived the immunity. HIV-positive children that are ART-naïve (antiretroviral therapy-naïve), usually have low CD4 counts, high viral loads and undernutrition [16]. In order to recognise risk factors associated with undernutrition, most studies have been conducted among HIV-positive children who are not yet on antiretroviral therapy. Therefore, no generalised findings to ART-treated HIV-positive children were published thus far [16].

The aim of this study was to describe the profile and clinical picture of admitted children with undernutrition to National District Hospital (NDH) in Bloemfontein, in order to identify areas for intervention. The objectives were to: describe the baseline demographics and anthropometric measurements of the children; identify the presenting symptoms, signs and diagnoses of the children; investigate if there is a difference in the presenting symptoms and signs of HIV-positive and HIV-negative children; investigate the nutritional intake of the children.

National District Hospital is a 197-bed district hospital located in Bloemfontein, the capital of the Free State province. The hospital is part of the academic training platform of the University of the Free State. National District Hospital is a referral hospital in the Mangaung area, however, many patients from surrounding towns without 24-hour health care services, as well as soft border towns present at the hospital for health care.

## Methods

This was a descriptive study with an analytical component. The study population consisted of all children from 2 to 71 months admitted to the Paediatric ward at NDH and diagnosed with undernutrition. This age category was chosen as children under two months are not routinely admitted in the setting and the definition used for undernutrition is applicable for children under six years. The study period from January 2016 to December 2017 was agreed upon to include the latest available data in accordance with ethical approval for this retrospective file review. Undernutrition was defined as the presence of one or more of the following as described by the World Food Programme (WFP) and the Centers for Disease Control and Prevention (CDC): [17] weight for age < -2 standard deviation (SD); height for age < -2 SD; presence of bilateral pitting oedema; micronutrient deficiency presenting with anaemia (haemoglobin (Hb) < 11 g/dl) in the absence of bleeding. This definition was used since information for all the necessary components was available in the patient files.

**Measurement:** data from the consultation notes and admission books available in the patient files were noted on a data sheet designed by the researchers from information in the literature. The following data were collected: demographic data, child´s primary caregiver, anthropometric data, presenting symptoms and signs, diagnosis, underlying diseases, HIV and tuberculosis results and the feeding history.

**Pilot study:** a pilot study was conducted at NDH in September 2018. The aim of the pilot study was to test the data sheet and to make arrangements to collect patient files. Data for the pilot study were collected from the files of ten children from the age of 2 to 71 months who were admitted to NDH with undernutrition. Minor adjustments were made to the data form, mainly to align the order of the questions with that of the admission books.

**Analysis of data:** data analysis was carried out by the department of Biostatistics at the University of the Free State. Results were summarised by frequencies and percentages (categorical variables). The presenting symptoms of HIV-positive and HIV-negative children were compared in 2x2 tables. All p-values < 0.05 were regarded as statistically significant.

**Ethical aspects:** approval was obtained from the Health Sciences Research and Ethics committee at the University of the Free State, as well as from the Free State Department of Health. All patient information was kept confidential and no person was identified in the study.

## Results

### The profile of children admitted

#### Inclusion in the study

During 2016 and 2017 a total of 1352 children from the ages of 2 to 71 months were admitted to NDH. Of all the children admitted to the hospital at that time, 237 children were classified as undernourished. Of those, 26 children were admitted more than once during the study period. Each child was only included once during the study, leaving 211 children that were classified as undernourished. Some children, however, had incomplete notes (7.6%), were transferred before admission (3.3%), or their patient files could not be found (7.6%). We, therefore, included 172 (81.5%) of the 211 eligible children in our study.

Most children qualified on more than one criterion for undernutrition (Table 1).

**Table 1:** percentage of children per inclusion criterion (n = 172)

Criterion	Percentage (%)
Weight for age < -2 SD	88.0
Length for age < -2 SD	51.8
Bilateral pitting oedema	18.6
Anaemia (Hb < 11g/dl)	34.9

Hb = haemoglobin; SD = standard deviation

### Demographic data

**Figure 1:**
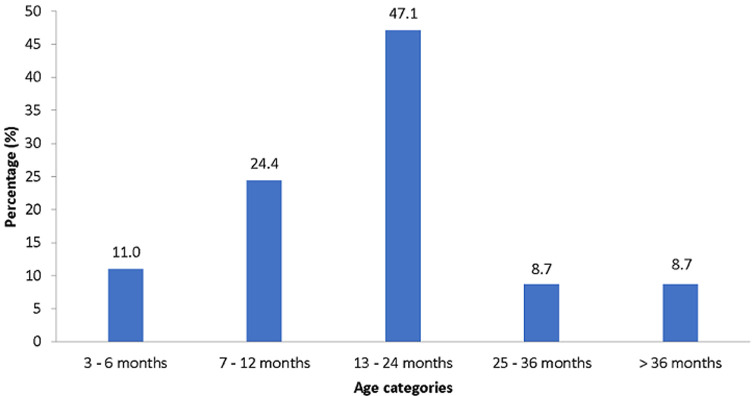
age distribution of children (n = 172) presented as a percentage

The gender distribution was almost equal with 51.2% males and 48.8% females. The ages varied from 3 months up to 63 months. The median age was 14 months. Eleven per cent of the children were under six months while 82.5% of children were under two years of age (Figure 1). Only 42.1% of admitted children were from Bloemfontein, while 57.9% came from rural areas. About 40% of the children (42.4%) had a low birth weight (< 2.5kg) while 28.6% of the children were born prematurely (< 37 completed weeks).

### Anthropometric data

**Figure 2:**
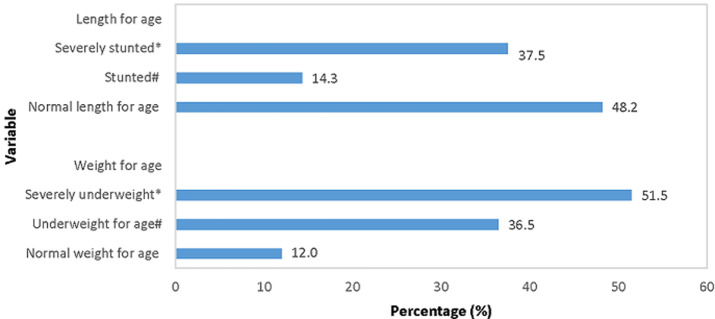
percentage of children per length and weight category according to age (n = 172). *Between -2 and -3 z-scores of the median WHO growth standards; #below -3 z-scores of the median WHO growth standards

Almost 90% of the children included in the study were underweight for age and more than half were stunted. The children were classified according to weight and length categories (Figure 2). The head circumference was only recorded for 16.3% of the children admitted with undernutrition. Head circumference of < -2SD was recorded for 35.7% of those with a recorded measurement.

### Presenting symptoms, signs and final diagnoses

The children presented with various symptoms. The children that presented with coughing include those who had pneumonia and upper respiratory tract infections. The children that presented with diarrhoea include those that had acute gastroenteritis and chronic diarrhoea. Most children had more than one presenting symptom, while four came for follow-up, without specific symptoms, but were admitted for various reasons. The major presenting symptoms were cough (45.7%), fever (35.8%), diarrhoea 46.3% and vomiting (43.8%). Feeding associated symptoms included: feeding problems (8.0%), swelling of the body (indicative of protein energy deficiency) (9.2%), and weight loss or not gaining weight (4.8%). The most prevalent presenting signs were anaemia (Hb <11g/dl) (34.9%), dehydration (20.4%), and enlargement of lymph nodes (18.6%). The final diagnoses as stated in the notes of the children are displayed in Table 2. Some children had more than one diagnosis.

**Table 2:** final diagnosis displayed as a percentage (n = 163)

Diagnosis	Percentage (%)
**Acute gastroenteritis**	30.0
**Pneumonia**	28.8
**HIV for treatment intervention**	21.5
**Anaemia**	36.8
**Tuberculosis**	21.5
**Severe acute malnutrition (SAM)**	55.8
**Moderate acute malnutrition**	14.1
**SAM with oedema**	14.1
**Other**	15.6

### Comparison of presenting symptoms and signs of HIV-positive and HIV-negative children

Only 21.7% of the children included in the study were HIV-positive of whom 82.9% were on antiretroviral treatment. There were no significant differences between the presenting symptoms of HIV-positive and HIV-negative children, with tiredness being close to significant. Only 17 (11.2%) of the children presented with tiredness: 20.6% of HIV positive children and 8.5% of HIV negative (p = 0.06).

In Table 3 the presence or absence of oedema were compared with HIV results. Oedema was present in 19.3% of the children and they were all HIV-negative or on antiretroviral treatment. No statistically significant differences were found for any of the presenting signs between HIV-positive and -negative children.

**Table 3:** oedema by HIV results

Frequency	HIV+	HIV-	Total
**No oedema**	30 (85.7%)	100 (79.4%)	130
**Oedema**	5^*^ (14.3%)	26 (20.6%)	31
**Total**	35	126	161

* All 5 these children were on antiretroviral treatment. Fisher´s exact test p-value=0.47

### Nutritional intake of the children

A detailed feeding history was recorded for less than 10% of the children, with no feeding history available for 11 (6.4%) of the children. Although 75.8% of children received breast milk at some stage, only 50% (4/8) of children under 6 months were currently breastfed and 37.5% were already on solids. Of all the children under 2 years of age 56.7% were currently receiving milk (3.2% mixed feeding, 44.4% breast milk and 52.4% formula feeding).

## Discussion

### The profile of children admitted

Since more than 80% of the undernourished admitted children over a two-year period were included, the results of this study can be generalised for admitted children with undernutrition in this hospital setting.

The majority (82.6%) of the children were under the age of two years. Children under two years of age are still in their first 1000 days of development. The first 1000 days of a child´s life, which starts at conception and ends at around the child´s second birthday, is crucial for the child´s development. In this period of life, foundations of optimal health, growth and neurodevelopment across the child´s lifespan, are established [18]. Undernourished children who are still in their first 1000 days of development also experience a significant reduction in brain development which cannot be regained later in life [19]. It is, therefore, crucial to identify and address undernutrition within the first 1000 days of life.

In total, 42.4% of the children had a low birth weight (< 2.5 kg), indicating that they were born with both a growth and developmental disadvantage. Of these undernourished children only 28.6% were born prematurely (< 37 completed weeks) where low birth weight was expected. A study performed in Bangladesh on 7530 low birth weight babies demonstrated a very strong link between low birth weight and undernutrition during childhood when all other contributing factors were controlled for [20]. In order to address undernutrition, low birth weight babies should be labelled as high risk for undernutrition and followed up diligently.

More than 50% of the undernourished children were from rural areas. In most of these areas primary health care services are only available from 08: 00 to 16: 00 on weekdays. The non-availability of 24-hour health care services in rural areas cause the caregivers to seek medical services elsewhere, which are usually far from home and in big centres like Bloemfontein. The problems with non-availability of 24-hour medical services were already highlighted in a 2009 study conducted in the same setting, where patients tend to access other medical services, thus compromising continuity of care [21]. Important factors in treatment protocols for undernutrition, like the continuity of care, poor follow-up and unavailability of support to caregivers, are almost impossible if children come from far away [22].

Stunting is an indication of long-term poor growth and is usually a combination of different factors. Danaei *et al*. identified 18 risk factors for stunting in children, which they grouped in five categories namely maternal nutrition and infection, teenage motherhood and short birth intervals, fetal growth restriction and preterm birth, child nutrition and infection, and environmental factors [23]. Underweight (weight for age < -2 SD) can be caused by acute episodes of weight loss or poor growth during episodes of illness or insufficient food intake or can present in combination with stunting indicating a more chronic problem [24]. In our study, 51.8% of the admitted undernourished children were stunted and 88% were underweight for age, indicating more acute or short term undernutrition. This may be an indication that undernutrition is picked up before stunting occurred. In South Africa, 29.1% of all children under 5 years are stunted [25]. In our setting, a study performed in primary health care facilities in Bloemfontein reported underweight for age in 7.7% of children under 5 years [26].

Growth monitoring and recording are part of routine care in primary health care facilities for all children up to five years of age. The head circumference was, however, not recorded for 83.7% of the children and for those recorded, 35.7% were smaller than normal, indicating poor brain growth. This highlights two important aspects: if the head circumference is not measured, poor brain growth will not be identified, and secondly, it will not be managed. As indicated earlier, brain growth is of utmost importance during the first 1000 days of life. Another focus for intervention should be on accurate and complete recording of anthropometric data, in order to identify children with undernutrition and intervene early.

### The presenting symptoms, signs and final diagnosis of undernourished children admitted

According to UNICEF, both disease and inadequate dietary intake cause undernutrition-the disease causes inadequate dietary intake, whereas inadequate dietary intake causes the disease. This is a vicious circle and interventions should be focused on disease as well as diet [27]. If a child is ill, he/she will not eat properly. Insufficient dietary intake may cause the immune system to weaken and can lead to undernutrition. On the other hand, a child can be undernourished, which causes the immune system to weaken that can further lead to infection and illness. From this study, the major presenting symptoms; namely cough, fever and diarrhoea, as well as the diagnosis of pneumonia, gastroenteritis, HIV, tuberculosis, anaemia and undernutrition are all covered in the Integrated Management of Childhood Illness (IMCI) programme [28]. Therefore, primary health care facilities should be able and equipped to manage children that present with the identified symptoms effectively. According to the IMCI guidelines, all children that present with any of the identified symptoms should be screened for nutritional status.

### Comparison of presenting symptoms and signs of HIV-positive and HIV-negative children

Many HIV programmes and guidelines address undernutrition as part of management. Children with HIV have a lower immune response and are more susceptible to opportunistic infections and chronic diarrhoea, which may contribute to undernutrition [29]. We could, however, not find a statistically significant difference in the presenting symptoms, signs or diagnosis of HIV-positive and HIV-negative children. A Ugandan study could also not find a difference in the presenting symptoms of HIV-positive and -negative children, except for oedema that was less likely in HIV-positive children not on antiretroviral treatment [30]. The presence of oedema was linked to higher CD4+ counts, fewer infections and older age children, rather than the HIV disease itself [30,31]. With the current low HIV transmission rates and the early initiation of antiretroviral treatment, HIV should not be a major contributor to undernutrition anymore; however, guidelines on nutritional interventions for immune-compromised children can be valuable resources in the management of undernutrition [32,33].

### Nutritional intake of the children

Of great concern is the 24.2% of children that were not breastfed and that only half of the children under two years currently receive any milk. According to the 2016 South African Demographic Health Survey (SADHS), 77% of children aged 6 to 23 months are not fed a minimum acceptable diet, and a large proportion of children in this age group are not breastfed [32]. The feeding data could, however, be inaccurate, since many patient files did not clearly indicate the feeding history. The data form also did not make provision for a detailed feeding history. Much more emphasis should be placed on a detailed feeding history during the assessment of children.

**Limitations of the study:** data were collected through a retrospective file review where all files were not available and only the recorded information could be used. The lack of a head circumference measurement and a detailed feeding history were notable.

## Conclusion

If undernutrition, or the risk for undernutrition, can be identified and addressed early in life, the consequences can be limited or rectified. From the baseline demographics and anthropometric measurements, the majority of children were under the age of two years, which meant that they were still in their first 1000 days of life, which is the crucial period for optimal brain growth and development For many of these children undernutrition already started in utero with almost half of the babies that were born with a low birth weight. No specific presenting symptoms or the HIV status were helpful indicators of undernutrition. However, the majority of the children presented with the symptoms and signs that are addressed in the IMCI and that will lead to the assessment of nutritional status if done correctly. Due to the lack of information in the files, the feeding history and nutritional intake could not be adequately assessed, however, a quarter of the children never received breastmilk. Obtaining a proper feeding history is crucial to address the specific feeding needs of children of different ages.

It is recommended that screening and intervention programmes for undernutrition should focus on the first 1000 days of life. Specific attention should be given to children with risk factors for undernutrition e.g. low birth weight, prematurity, lack of breastfeeding and residence in rural areas. Further, nurses in primary health care clinics should be able to identify undernutrition if they follow the presenting symptoms and nutritional assessment guidelines. All anthropometric data, including head circumference, should be recorded and proper feeding advice should be included in all assessment and management of children.

### What is known about this topic

- The first 1000 days of life is crucial for brain development;
- Nutrition plays a major role in brain development;
- Undernutrition is very common in Africa.

### What this study adds

- Anthropometric measurements are not routinely done at primary health care and therefore undernutrition is missed;
- Undernourished children present with the same symptoms and signs as well-nourished children;
- It is important to monitor children at risk for undernutrition diligently and intervene early.
